# Stability of Microbial Community Profiles Associated with Compacted Bentonite from the Grimsel Underground Research Laboratory

**DOI:** 10.1128/mSphere.00601-19

**Published:** 2019-12-18

**Authors:** Katja Engel, Sian E. Ford, Sara Coyotzi, Jennifer McKelvie, Nikitas Diomidis, Greg Slater, Josh D. Neufeld

**Affiliations:** a Department of Biology, University of Waterloo, Waterloo, Ontario, Canada; b School of Geography & Earth Sciences, McMaster University, Hamilton, Ontario, Canada; c Nuclear Waste Management Organization, Toronto, Ontario, Canada; d NAGRA, Wettingen, Switzerland; University of Wisconsin—Madison

**Keywords:** MX-80, bentonite, clay, nuclear waste disposal, microbial characterization, 16S rRNA gene sequencing, PLFA, nuclear waste storage

## Abstract

To assess the microbiology and corrosion potential of engineered components of a deep geological repository for long-term storage of high-level nuclear waste, the Materials Corrosion Test is being conducted at the Underground Research Laboratory in Grimsel, Switzerland. Modules containing metal coupons surrounded by highly compacted MX-80 bentonite, at two dry densities (1.25 and 1.50 g/cm^3^), were emplaced within 9-m-deep boreholes, and the first modules were retrieved after 13 months of exposure. Bentonite and associated module materials were sampled, and microbial communities and their distributions were assessed using 16S rRNA gene sequencing and phospholipid fatty acid (PLFA) analysis. Borehole fluid was dominated by amplicon sequence variants (ASVs) affiliated with *Desulfosporosinus* and *Desulfovibrio*, which are putatively involved in sulfate reduction. The relative abundance of these ASVs was lower for samples from inside the borehole module, and they were almost undetectable in samples of the inner bentonite layer. The dominant ASV in case and filter sample sequence data was affiliated with Pseudomonas stutzeri, yet its relative abundance decreased in the inner layer samples. *Streptomyces* sp. ASVs were relatively abundant in all bentonite core sample data both prior to emplacement and after 13 months of exposure, presumably as metabolically inactive spores or extracellular “relic” DNA. PLFA concentrations in outer and inner layer bentonite samples suggested cellular abundances of 1 × 10^6^ to 3 × 10^6^ cells/g, with similar PLFA distributions within all bentonite samples. Our results demonstrate consistent microbial communities inside the saturated borehole module, providing the first evidence for microbial stability under conditions that mimic a deep geological repository.

**IMPORTANCE** The Materials Corrosion Test in Grimsel Underground Research Laboratory, Switzerland, enables an evaluation of microbiological implications of bentonite clay at densities relevant for a deep geological repository. Our research demonstrates that after 13 months of exposure within a granitic host rock, the microbial 16S rRNA gene signatures of saturated bentonite clay within the modules were consistent with the profiles in the original clay used to pack the modules. Such results provide evidence that densities chosen for this emplacement test are refractory to microbial activity, at least on the relatively short time frame leading to the first time point sampling event, which will help inform *in situ* engineered barrier system science. This study has important implications for the design of deep geological repository sites under consideration for the Canadian Shield.

## Abstract (precis)

The Materials Corrosion Test in Grimsel Underground Research Laboratory, Switzerland, enables an evaluation of microbiological implications of bentonite clay at densities relevant for a deep geological repository. Our research demonstrates that after 13 months of exposure within a granitic host rock, the microbial 16S rRNA gene signatures of saturated bentonite clay within the modules were consistent with the profiles in the original clay used to pack the modules. Such results provide evidence that densities chosen for this emplacement test are refractory to microbial activity, at least on the relatively short time frame leading to the first time point sampling event, which will help inform *in situ* engineered barrier system science. This study has important implications for the design of deep geological repository sites under consideration for the Canadian Shield.

## INTRODUCTION

The Nuclear Waste Management Organization (NWMO) is responsible for implementing an adaptive phased management plan for the long-term care of used nuclear fuel produced by Canada’s nuclear reactors. In the current design, high-level radioactive waste will be isolated within a deep geological repository (DGR) constructed at an approximately 500-m depth in low-permeability host rock in a willing and informed community (1). The DGR includes an engineered barrier system consisting of used fuel containers surrounded by highly compacted bentonite clay. The used fuel container will be coated with 3 mm of copper applied directly by cold spray and electrodeposition onto a carbon steel container that holds 48 Canada Deuterium Uranium (CANDU) fuel bundles. The steel provides the used fuel container with strength, whereas copper is for corrosion protection (2, 3). Under anoxic conditions, sulfate-reducing bacteria (SRB) in the repository have the potential to produce sulfide, which could diffuse through the highly compacted bentonite to the container and cause microbiologically influenced corrosion (3). However, bentonite swelling pressure and low water activity reduce the activity and survival of microorganisms (4–8). Previous research demonstrated that growth of bacteria and germination of spores did not occur when Wyoming MX-80 bentonite dry densities exceeded 1.6 g/cm^3^, water activity decreased below 0.96, or swelling pressure exceeded 2 MPa (4, 6). In addition to protecting the container from microbiologically influenced corrosion, highly compacted bentonite provides mechanical support to the container, retains radionuclides in the event of container failure, and provides a thermally conductive medium to transmit heat to surrounding host rock (9).

The NWMO is participating in the Materials Corrosion Test (MaCoTe) at the Grimsel Underground Research Laboratory (URL) in collaboration with international nuclear waste agencies. The Grimsel test site is situated in the Swiss Alps, in granitic rock. Test modules containing candidate materials for used fuel containers are embedded in bentonite and emplaced within 9-m-deep boreholes. Modules are saturated and exposed to native groundwater, currently for a duration of up to 9 years, with the retrieval of modules occurring at various time intervals. Once retrieved, samples of candidate materials and bentonite will be analyzed with a full suite of corrosion and microbiological tests to evaluate corrosion performance and the ability of bentonite to suppress microbial activity. The main goals of the MaCoTe program are to confirm the long-term corrosion rate of candidate canister materials in compacted bentonite under anoxic repository-relevant conditions and to provide experimental evidence for the inhibitory effects of the bentonite buffer on microbial activity and microbiologically influenced corrosion.

In this study, both high-throughput sequencing of 16S rRNA genes and phospholipid fatty acid (PLFA) analysis were used to characterize microbial communities associated with bentonite from the first test modules that were retrieved after 13 months of *in situ* exposure. The results of this first time point analysis, focused on modules with bentonite clay at two dry densities (1.25 and 1.50 g/cm^3^), demonstrate the relative stability of internal module microbial profiles and serve as important baseline data for comparison to additional modules that will be recovered in subsequent years.

## RESULTS AND DISCUSSION

### Microbial 16S rRNA gene profiles.

**FIG 1:**
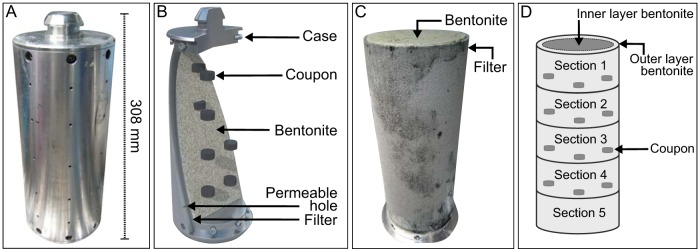
Schematic of the borehole module design. (A) Image of the borehole module showing the case and diffusive holes. (B) Schematic showing the borehole module with metal coupons embedded in bentonite surrounded by a sintered stainless steel filter and metal case. (Modified from Necib et al. [31].) (C) Image of the filter with compacted bentonite inside. (D) Schematic of the bentonite core showing five sections and location of “inner” and “outer” layers of bentonite. Section 5 of the bentonite core does not contain coupons. The outer and inner layers of section 5 were sampled separately. The outer layer refers to the approximately 5-mm-thick outer ring of bentonite around the core.

Stainless steel borehole modules with corrosion test pieces embedded in bentonite (Fig. 1) were incubated under anoxic conditions in a 9-m-deep borehole at the Grimsel URL. After 13 months of exposure, modules 1A (1.25 g/cm^3^) and 2A (1.50 g/cm^3^) were recovered from borehole 13.001 in October 2015. Total genomic DNA was extracted from swabs, bentonite, and Sterivex filters (see Table S1 in the supplemental material). In addition to the borehole module samples, we also analyzed pellets and powdered Wyoming MX-80 bentonite that was used to load the modules before exposure in the borehole. Depending on sample type, the PowerSoil or PowerMax DNA isolation kits were used, as previously validated for clay samples (10). Although only 18 DNA extractions yielded sufficient amounts of genomic DNA to be quantified using the Qubit fluorometer, 42 samples yielded an amplicon in nested 16S rRNA gene PCR (Table S1); however, many PCR products were only weakly visible by agarose gel electrophoresis (data not shown). Reagent and laboratory contaminant sequences can contribute a large proportion of detectable DNA in samples associated with low biomass. As a result, careful reagent and workspace decontamination was performed prior to PCR analysis. Sequencing results were examined to differentiate sample-specific signal from “noise” (see supplemental material). Sequencing of the borehole module samples generated 1,152,594 paired-end reads, which were assigned to 1,589 bacterial and archaeal amplicon sequence variants (ASVs).

10.1128/mSphere.00601-19.1 TABLE S1 List of controls and samples from borehole modules. DNA was extracted using the PowerSoil (PS) or PowerMax (PM) DNA isolation kit. Genomic DNA (gDNA) concentrations were determined using the Qubit dsDNA High Sensitivity assay kit. Samples with concentrations below detection limit are indicated with BDL. The success of the nested PCR (PCR2, total of 50 cycles) was summarized stating the presence (yes) or absence (no) of an amplicon on an agarose gel. The 16S rRNA gene was amplified from DNA extracts using primer pair 1 or 2 as indicated. Phospholipid fatty acids (PLFAs) were analyzed from selected samples as indicated. Download Table S1, PDF file, 0.2 MB. Copyright © 2019 Engel et al. 2019 Engel et al. This content is distributed under the terms of the Creative Commons Attribution 4.0 International license.

### Microbial diversity in borehole module samples.

**FIG 2:**
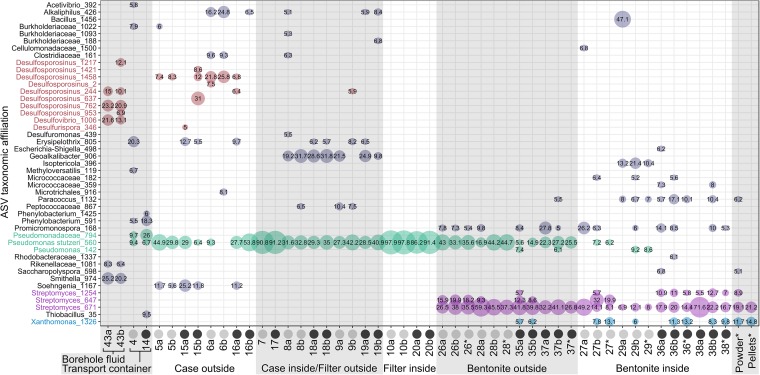
Microbial community composition of borehole module samples after 13 months of exposure. A mixture of pellets and powdered bentonite was used to prepare borehole modules 1A (gray circles) and 2A (black circles) at dry densities of 1.25 and 1.50 gm/cm^3^, respectively. After assembly, the bentonite was fully saturated using pore water extracted from borehole 13.001, and modules were placed in borehole at the Grimsel URL in Switzerland. After 394 days of exposure, the modules were retrieved and analyzed. Samples are sorted from left to right by location within the module, from outside to inside. Results of two replicate DNA extractions (a and b) using the PowerSoil DNA isolation kit or PowerMax DNA isolation kit are shown. No replicates are shown for samples 4, 7, 14, and 17. *, samples that were amplified using primer 515F-Y/926R. All other samples were amplified using Pro341F/Pro805R. Relative abundances of microbial taxa (≥6% abundance) are shown. Colors correspond to taxa that are discussed in the text.

**FIG 3:**
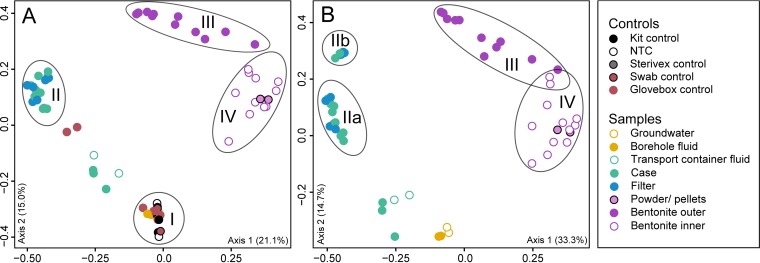
Grouping of borehole module samples in a PCoA ordination based on Bray-Curtis metrics. PCoA plots were generated including (left) or excluding (right) controls. Ordinations were rarefied to 500 (A), or 2,000 (B) reads per sample. All controls are contained in group I, except two glove box controls from the last day of disassembly. Groups II, IIa, and IIb contain the majority of case and filter samples and separated well from outer (group III) and inner (group IV) bentonite core samples.

The 16S rRNA gene sequence data showed distinct taxonomic profiles (Fig. 2) and location-specific grouping of samples from the outer to the inner regions of the borehole modules (Fig. 3). Controls separated well from bentonite and filter samples, as well as the majority of case samples (Fig. 3A). The principal-coordinate analysis (PCoA) plots showed that microbial communities in the bentonite core differed from all case and filter samples as well as from borehole fluid (Fig. 3B). Fluid from borehole 13.001 was sampled using a Sterivex filter, and 150 ng DNA was recovered, which was the largest amount of DNA extracted from any sample in this study (Table S1). A total of 27 ASVs were identified in the borehole fluid, and the most abundant ASVs were affiliated with *Desulfosporosinus*, *Smithella*, and *Desulfovibrio* (Fig. 2); these ASVs were absent from controls (CTRL7 and 8) (see Fig. S1). Characterized members of both *Desulfosporosinus* and *Desulfovibrio* are anaerobic sulfate-reducing bacteria (11). Known members of the genera *Smithella* are strictly anaerobic, degrading fatty acids and aromatic acids in syntrophic association with methanogens or sulfate reducers (12, 13). The relative abundance and potential activity of all three abundant ASVs were reduced greatly in samples taken from inside the borehole module and were essentially absent from the inner layer of bentonite (Fig. 2).

10.1128/mSphere.00601-19.6 FIG S1 Bubble plot showing 16S rRNA gene profiles of controls and borehole module samples. Results of no-template controls (NTCs), kit controls (simulated DNA extraction from kit buffer instead of a sample), swab controls (simulated DNA extraction from unused swabs), and Sterivex controls (simulated DNA extraction from unused Sterivex filters) are shown. For a simplified view, read counts for replicate NTCs, swab controls, Sterivex controls, and MaCoTe samples were pooled into one composite. Modules were placed into borehole 13.001 at Grimsel URL, which contained natural groundwater retrieved from a neighboring borehole 85.003. Swab samples were taken from case and filter of module 1A (M1aCase) and module 2A (M2aCase). Bentonite samples were analyzed from module 1A (M1aBentonite) and 2A (M2aBentonite). The plot is based on unrarefied data, and read counts for each sample (or composite) are shown at the end of the sample name. ASV taxonomic affiliation is shown on the *y* axis followed by an ASV number. Only ASVs at or above 5% relative abundance are shown. Download FIG S1, PDF file, 0.5 MB. Copyright © 2019 Engel et al. 2019 Engel et al. This content is distributed under the terms of the Creative Commons Attribution 4.0 International license.

The DNA extract from shipping flask liquid of module 2A was dominated by an ASV affiliated with *Phenylobacterium*, which is a Gram-negative and strictly aerobic organism from the *Caulobacteraceae* family. For module 2A, 18.3% of all reads belonged to this ASV, in contrast to a relative abundance of 5.5% in module 1A (Fig. 2). No reads associated with this ASV were identified in the fluid of borehole 13.001 (see ASV Table 1 posted at https://doi.org/10.5281/zenodo.3530762), which was sampled after recovery of the modules and frozen at −20°C. The shipping flask fluid was sampled during disassembly of the modules in the glove box, approximately 20 days after retrieval. However, the transfer flask appeared to be anoxic based on oxygen monitors within the glove box. The origin of *Phenylobacterium* is unclear, yet introduction during packing of the modules is possible.

Across all case and filter samples, the dominant ASV was affiliated with Pseudomonas stutzeri (Fig. 2), which was absent from both kit controls (CTRL1 and 2) (Fig. S1). During disassembly of modules 1A and 2A, small black spots (<0.5-cm diameter) were visible on the stainless steel filter as well as on the surface of the bentonite core (Fig. S1). A ring of a black deposit was found on the inside of the case lid for both modules (see Fig. S2B). The 16S rRNA gene sequence data revealed that ∼90% of the reads in both samples from black deposit were affiliated with Pseudomonas stutzeri (samples 7 and 17) (Fig. 2). Strains of Pseudomonas stutzeri are capable of producing an extracellular black-colored pigment (14), which might be the cause for the black spots visible in the borehole module. However, sulfate-reducing bacteria are also reported in MX-80 bentonite (15), and black sulfide deposits on bentonite or copper surfaces were seen previously (7, 8, 16–18). We did not perform a chemical analysis of the material but recommend this for subsequent module sampling events.

10.1128/mSphere.00601-19.7 FIG S2 Photographs of borehole module 1A after 13 months of exposure to Grimsel URL pore water. (A) Outside view of the borehole model (case) with white arrow pointing to a permeable hole. (B) View of the inside surface of the case lid. White arrow points to a ring of black deposit. (C) View of the opened case with the filter and bentonite core inside. Black deposit can be seen at the outer edge of the core. (D) View of the outside surface of the stainless steel filter with the bentonite core inside. Black stained areas can be seen on the filter surface. (E) View of the inside surface of the stainless steel filter after the bentonite core was removed. Black spots can be seen on several locations on the surface. (F) View of the outside surface of the bentonite core. Black spots can be seen on several locations. The white circle in images E and F point to the colocalization of spots on the filter and bentonite surface. The location of the black spots on the bentonite core do not colocalize with the diffusive holes in the metal case. Download FIG S2, PDF file, 2.9 MB. Copyright © 2019 Engel et al. 2019 Engel et al. This content is distributed under the terms of the Creative Commons Attribution 4.0 International license.

In samples of pellets and powdered Wyoming MX-80 bentonite that were used to pack the borehole modules, we identified dominant ASVs affiliated with *Streptomyces* and *Xanthomonas* (Fig. 2). Both ASVs were also present in the inner bentonite core after 13 months of exposure (Fig. 2). The microbial communities of inner bentonite samples appeared to be very similar to those in the original MX-80 material (Fig. 3B), suggesting that those microorganisms were present before the start of the 13-month incubation. The presence of *Streptomyces* in the bentonite clay indicates the resistance of these taxa to the targeted swelling pressures, presumably in the form of metabolically inactive spores or extracellular “relic” DNA. Before starting DNA isolation from inner-layer bentonite samples, we removed several millimeters of bentonite from the outside using a sterile, DNA-free single-use scalpel to remove potential contamination from the outer layer of bentonite, filter, and case. Therefore, the dominance of ASVs affiliated with *Streptomyces* in the bentonite core is unlikely due to contamination. Indeed, Persson et al. (19) identified Streptomyces chungwhensis and Streptomyces monomycini in MX-80 bentonite, with a density of 1,850 g/cm^3^, and Chi Fru and Athar (20) identified Streptomyces albidoflavus in bentonite with a density of 2,000 g/cm^3^. *Streptomyces* spp. are considered aerobic organisms and thus unlikely to grow in an anoxic repository. As a result, the detection of *Streptomyces* DNA within bentonite clay associated with the MaCoTe project and for future module samples will provide confidence in the absence of bacterial growth within the inner bentonite clay regions of the modules. Combining 16S rRNA gene sequencing and quantitative approaches, such as quantitative PCR, with or without spiking all samples with known quantities of reference DNA (21), would help assess changes in bacterial abundances for analysis of samples in the future.

As mentioned previously, Pseudomonas stutzeri was the dominant ASV across all case and filter samples. The ASV was also associated with 5.6% to 44.7% of reads in the outer-layer bentonite (Fig. 2). The highly compacted bentonite core is thought to prevent microbial growth (22), and the relative abundance and potential activity of Pseudomonas stutzeri were reduced when moving toward the inside of the borehole module (Fig. 2). On average, only 2.8% of reads were associated with Pseudomonas stutzeri in the inner bentonite of module 1A, and no reads matched this species in module 2A (ASV Table 1). Although nested PCR and variable cycle numbers may generate artifacts and affect relative abundances of detected taxa, amplification of bentonite samples for 35, 45, and 50 cycles showed highly similar microbial community profiles (see Fig. S3). Because our analyses only measured relative abundances, the observed decrease of *Pseudomonas* in the bentonite core can also be due to an increase in the numbers of sequences from other genera in the inner bentonite layer. However, the distribution of abundant operational taxonomic units (OTUs) between inner and outer layer bentonite are relatively similar, and only *Pseudomonas* can be identified with a strong shift in relative abundance (Fig. 2). Pseudomonas stutzeri is a Gram-negative, motile, and denitrifying or fermentative bacterium that is commonly found in MX-80 bentonite (19, 20, 23, 24). Indeed, *Pseudomonas* spp. can comprise up to 26% to 35% of the microbial community in borehole water that was maintained under anoxic conditions for 10 months, presumably growing fermentatively on organic matter from clay and releasing organic acids and hydrogen (25). ASVs associated with Pseudomonas stutzeri were not present in the groundwater (borehole 85.003) or in borehole 13.001 microbial community (ASV Table 1). For future sampling and analysis of MaCoTe samples, the presence of *Pseudomonas* sp. sequences will be used as a measure of microbial growth associated with anthropogenic disturbance related to borehole module emplacement.

10.1128/mSphere.00601-19.8 FIG S3 Effect of various PCR cycle numbers on microbial community distributions and relative abundances. The 16S rRNA gene profiles (A) and grouping of bentonite samples in a PCoA ordination based on Bray-Curtis metrics (B) after 35, 45, or 50 (nested) cycles of PCR amplification. (A) Only ASVs at or above 2% relative abundance are shown. (B) Samples were rarefied to 6,000 reads. Sequences were analyzed using QIIME2 (release 2019.7) with primer sequences removed using DADA2 (version 1.6) and low-quality bases trimmed after 241 and 205 nucleotides (nt) for forward and reverse reads, respectively. Taxonomy was assigned to ASVs using a Naive Bayes classifier (feature-classifier classify-sklearn) pretrained with SILVA database release 132 with reference sequences trimmed to the target region (Pro341F/Pro805R) to improve taxonomic classification (41). Download FIG S3, PDF file, 0.3 MB. Copyright © 2019 Engel et al. 2019 Engel et al. This content is distributed under the terms of the Creative Commons Attribution 4.0 International license.

Microbial activity and cell survival are known to decrease with higher bentonite clay compaction and higher swelling pressures (5–8). In borehole modules 1A and 2A, bentonite was compacted targeting 1.25 and 1.50 g/cm^3^ dry density, respectively, and we used ASV richness as a proxy of how many species are present in the bentonite. However, ASV richness in the inner layer bentonite samples of module 2A did not differ significantly from those in module 1A (Table S2) nor did the community composition (Fig. 2). The 16S rRNA gene sequencing data showed grouping of replicate samples, especially for the outer-layer bentonite samples. Inner-layer bentonite samples from both borehole modules did not form groups distinct from each other (see Fig. S4). Although 16S rRNA gene sequencing determined the total microbial community in the bentonite samples, the presence of these taxa with sequence data does not necessarily reflect activity or viability. Although RNA abundances can be better used as a proxy for an active microbial community, RNA test extractions on module 1A inner-layer bentonite (number 27) were unsuccessful.

10.1128/mSphere.00601-19.2 TABLE S2 ASV counts in inner and outer bentonite samples. Data were rarefied to 3,300 reads per sample. ASV counts are shown for full dataset and for ASVs at or above 1% relative abundance. Download Table S2, PDF file, 0.1 MB. Copyright © 2019 Engel et al. 2019 Engel et al. This content is distributed under the terms of the Creative Commons Attribution 4.0 International license.

10.1128/mSphere.00601-19.9 FIG S4 Grouping of inner- and outer-layer bentonite samples from the borehole modules 1A and 2A in a PCoA ordination based on Bray-Curtis metrics. Bentonite was compacted targeting 1.25 (1A) and 1.50 (2A) gm/cm^3^ dry density. Samples were rarefied to 3,300 reads per sample. Replicate extractions of the same bentonite sample were amplified with Pro341F/Pro805R (a and b) or 515F-Y/926R (*). Download FIG S4, PDF file, 0.1 MB. Copyright © 2019 Engel et al. 2019 Engel et al. This content is distributed under the terms of the Creative Commons Attribution 4.0 International license.

### Minor variations in lipid profiles among bentonite samples.

**FIG 4:**
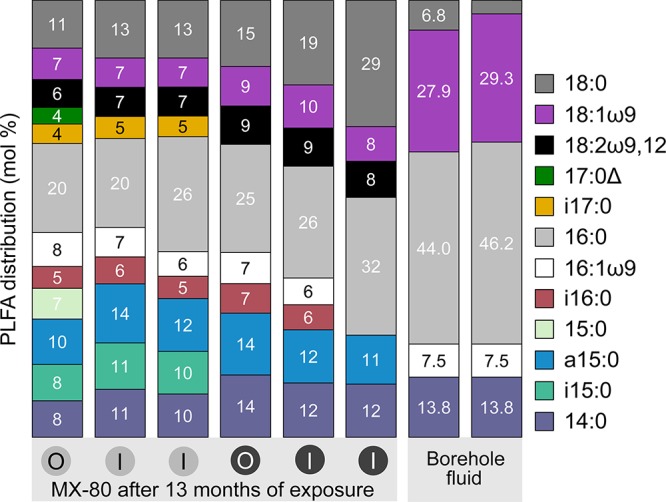
PLFA distributions in bentonite samples from borehole modules 1A and 2A and borehole 13.001 fluid. Bentonite from the outer (O) and inner (I) layers of the bentonite cores of borehole module 1A (gray circles) and 2A (black circles) were analyzed after 13 months of exposure. Fluid from borehole 13.001 was sampled on the day of removal of the modules using a Sterivex filter. The PLFA terminology of A:BωC,D indicates total number of carbon atoms (A), number of double bonds (B), number of carbon atoms between the double bond(s), and the aliphatic end of the molecule (C and D). Δ, a cyclo-substituted PLFA.

Inner- and outer-layer bentonite samples from modules 1A and 2A were analyzed for PLFA abundances and distributions after 13 months of exposure in the borehole. Only 12 individual PLFAs were present in the bentonite core samples (Fig. 4), and profiles for both modules were dominated by general bacterial markers (i.e., 14:0, 16:0, and 18:0) (26). The PLFA-based cellular abundance estimates (27) were similar for both modules, with 1 × 10^6^ to 3 × 10^6^ cells/g, indicating no significant differences between low- and high-density bentonite modules (see Table S3).

10.1128/mSphere.00601-19.3 TABLE S3 PLFA concentrations and estimated cellular abundances based on generic conversion factor (Green and Scow [27]). NA, not applicable. Download Table S3, PDF file, 0.1 MB. Copyright © 2019 Engel et al. 2019 Engel et al. This content is distributed under the terms of the Creative Commons Attribution 4.0 International license.

Based on 16S rRNA gene profiles, the bentonite cores of both modules were dominated by an ASV associated with *Streptomycetaceae*. The representative sequence of this ASV had 100% identity with Streptomyces iconiensis and Streptomyces chumphonensis (see Table S4). Under cultured conditions, the major cellular fatty acids in both organisms are *anteiso* C_15:0_ (22.0% to 46.5%), *iso* C_16:0_ (13.3% to 22.7%), *anteiso* C_17:0_ (11.2% to 18.2%), and *iso* C_15:0_ (10.0% to 14.2%) (28, 29). The presence of *anteiso* C_15:0_ and *iso* C_16:0_ in both modules could be consistent with the presence of *Streptomyces.* Although modules 1A and 2A showed very similar PLFA distributions (Fig. 4), *iso* C_15:0_ and *iso* C_17:0_ were only present in module 1A and can be indicators for Gram-positive bacteria (26). The detection of C_18:2_ω_9,12_ can be indicative of fungi (26, 30). The 16S rRNA gene analysis did not target this group of microorganisms.

10.1128/mSphere.00601-19.4 TABLE S4 Result of NCBI BLASTN (v 2.8.1) (44) of ASV number 671 representative sequence against 16S ribosomal RNA (*Bacteria* and *Archaea*) database. Download Table S4, PDF file, 0.1 MB. Copyright © 2019 Engel et al. 2019 Engel et al. This content is distributed under the terms of the Creative Commons Attribution 4.0 International license.

### Conclusions.

We demonstrate relatively stable microbial community profiles associated with bentonite clay at two dry-density swelling pressures following 13 months of subsurface emplacement. Because we detected Pseudomonas stutzeri in disturbed samples associated with the outer surfaces of the borehole module, we suggest that these bacteria could serve as indicators of microbial growth external to the bentonite clay modules. In contrast, bentonite-specific microbial signatures dominated by *Streptomyces* sp. taxa were consistent after subsurface emplacement, regardless of swelling pressure, suggesting little microbial growth by the first sampling time point. Given that this is a 10-year experiment, future sampling efforts will determine whether microbial community stability and presumed inactivity persists. The current research also further validates a protocol for DNA-based analysis of low-biomass bentonite clay samples, leveraging extraction controls to confirm sample-specific microbial signatures. Future research will ideally incorporate cultivation and microbial activity assays to help confirm microbial viability in the bentonite cores of the borehole modules.

## MATERIALS AND METHODS

### Experimental setup.

Stainless steel borehole modules and the arrangement of corrosion test pieces were described previously (31, 32). Assembly of Materials Corrosion Test (MaCoTe) modules was performed under anoxic conditions. Corrosion coupons (20-mm diameter) were manufactured from carbon steel (10-mm height), cold spray copper (3-mm height), electrodeposited copper (3-mm height), wrought copper (10-mm height), and stainless steel (4-mm height). A mixture of pellets and powdered Wyoming MX-80 bentonite was used to prepare modules with dry densities of 1.25 (module 1A) and 1.50 g/cm^3^ (module 2A). After assembly of the modules, the bentonite was fully saturated in pore water extracted from borehole 13.001 and saturated for 10 days within the anaerobic chamber. A sample of the borehole fluid used to saturate the module or at time of emplacement was not analyzed. Modules were sealed into 2 to 3 layers of argon gas-filled Mylar bags and transported to the Grimsel URL. During transport, bags of modules 1A and 2A were damaged and may have been exposed to the atmosphere. A total of 8 modules were placed into a 9-m-deep vertical borehole (13.001) at Grimsel URL on 22 September 2014. Diffusive holes in the modules permitted exchange with the environment (Fig. 1). After placement of the modules, the borehole was flushed with argon and sealed with a hydraulic packer to maintain anoxic conditions as described previously (31, 32). Modules will be removed and analyzed according to a schedule over a 10-year period.

### Removal and sampling of borehole modules.

On 21 October 2015, after 13 months (394 days) of exposure at the Grimsel URL, two modules were removed from borehole 13.001 while purging with argon gas. The modules were placed into individual stainless steel transfer flasks and anoxic conditions were maintained. Fluid from borehole 13.001 was sampled on the day of removal using a Sterivex-GP 0.22-μm filter (Millipore, MA, USA) and stored at −20°C until analysis. Modules were transported to AMEC Foster Wheeler laboratories (Harwell, UK) for sampling in an anoxic glove box as previously described (31, 32). Disassembly was performed 10 to 14 November 2015. No spike in oxygen concentration was observed when opening the transfer flasks in the glove box, verifying anoxic conditions during shipment and storage. Liquid from the bottom of the shipping flask was aspirated using a sterile syringe, transferred to a sterile 50-ml tube, and stored at −20°C until analysis. Knives for cutting and sampling were flame sterilized with 70% ethanol followed by heat treatment to remove DNA and wrapped in sterile aluminum foil before transferring into the anaerobic chamber. Bentonite cores were cross-sectioned approximately 1 cm below each layer of coupons, resulting in five sections (Fig. 1D). After sectioning, the outer layer of each bentonite section was sampled (approximately the outer 0.5 cm bentonite) before sampling the inner core. Further samples for microbiological analysis included swabs from the borehole modules and a detailed list of samples can be found in Table S1 in the supplemental material. All samples were frozen at −20°C until analysis. AMEC Foster Wheeler retrieved and analyzed metal coupons embedded in the bentonite, and results will be reported elsewhere. Here, we report the 16S rRNA gene and PLFA analysis of bentonite and other environmental samples related to MaCoTe borehole modules 1A and 2A.

### DNA extraction.

Total genomic DNA from sterile DNA-free foam swabs (Puritan, ME, USA) was extracted using the PowerSoil DNA isolation kit (MO BIO Laboratories, CA, USA). The swab tip was cut with flame-sterilized and flame-heated scissors into the PowerBead tube. After addition of lysis solution, the PowerBead tube was incubated at 70°C for 10 min followed by bead beating using a FastPrep-24 instrument (MP Biomedicals, OH, USA) at 5.5 m/s for 45 s. The remainder of the extraction was carried out according to the manufacturer’s instructions. Purified DNA was eluted into 60 μl of 10 mM Tris and stored in aliquots at −20°C until PCR amplification. Ten milliliters of transport container fluid was centrifuged at 7,000 × *g* for 15 min. The pellet was resuspended using liquid from bead beating tubes. The remainder of the extraction was carried out as described above. Sterivex housings were opened with flame-sterilized and flame-treated pliers. Filter membranes were removed using a sterile, DNA-free single-use razor blade. One quarter of each filter was used for extraction with the PowerSoil DNA isolation kit (MO BIO Laboratories) as described above. DNA from compacted bentonite was extracted using the PowerMax DNA isolation kit (MO BIO Laboratories). Several millimeters of bentonite were removed from inner-layer samples using a sterile and DNA-free single-use scalpel to remove potential contamination from sampling equipment or from the outer layer of bentonite. All samples were cut into small pieces before placing into the bead beating tube to aid suspension in the extraction buffer. A total of 2 g of inner- or outer-layer bentonite was added to PowerBead tubes, and after addition of PowerBead and lysis solution, the tube was incubated at 65°C for 30 min before bead beading for 10 min at 30 Hz (Mixer Mill MM 400; Retsch, Germany). The remainder of the extraction was carried out according to the manufacturer’s instructions. Purified DNA was eluted in 2 ml of 10 mM Tris. Nucleic acids were precipitated using 8 μl of Co-Precipitant linear polyacrylamide (Bioline, Germany), 0.1 volumes of 5 M NaCl (American Chemical Society [ACS] grade, prepared in molecular biology-grade water and filtered through a 0.2-μm syringe filter), and 1 volume of isopropanol (high-performance liquid chromatography [HPLC] grade) and stored at −20°C overnight. The DNA was pelleted by centrifugation at 13,000 × *g* for 30 min and then washed with 80% ethanol (HPLC grade), air dried at room temperature (5 to 10 min), and eluted in 120 μl of elution buffer. Aliquots were frozen at −20°C until PCR analysis. Genomic DNA was quantified using the Qubit dsDNA High Sensitivity assay kit (Invitrogen, CA, USA) with fluorescence measured on a Qubit 2.0 fluorometer (Life Technologies, CA, USA).

Due to the low biomass in samples and the high risk for contaminants to influence sample signals, replication and inclusion of controls were essential for this study. Extraction blanks were prepared with each batch of PLFA and DNA extraction (Table S1). No-template controls were prepared with samples processed for 16S rRNA gene amplification and included with the sequencing of samples, even if control amplicons are not detected. Several DNA extraction “kit controls” (simulated DNA extraction from kit buffer instead of a sample), “swab controls” (simulated DNA extraction from an unused swab), and Sterivex control (simulated DNA extraction from an unused Sterivex filter) were included. Controls for the DNA isolation kit reagent (kit control) were performed with each sample extraction batch.

### Amplification of 16S rRNA genes and sequencing.

The V3-V4 regions of 16S rRNA genes were amplified using universal prokaryotic primers Pro341F and Pro805R (33). Each primer contained a unique six-base index sequence for sample multiplexing as well as Illumina flow cell binding and sequencing sites (34). The 25-μl PCR mix contained 1× ThermoPol buffer, 0.2 μM forward primer, 0.2 μM reverse primer, 200 μM deoxynucleoside triphosphates (dNTPs), 15 μg bovine serum albumin (BSA), 0.625 U *Taq* DNA polymerase (New England Biolabs, MA, USA), and up to 10 ng template. Each PCR was prepared in triplicates and in two rounds. The first round PCR (PCR1) was performed as follows: 95°C for 3 min, 35 cycles of 95°C for 30 s, 55°C for 30 s, and 68°C for 1 min, and a final extension of 68°C for 7 min. A second round of PCR (PCR2 or “nested PCR”) was performed using 1 μl of template from PCR1 and amplified as described above for 15 cycles. Equal quantities of nested PCR amplicons were pooled to a maximum of 30 μl. Controls (no-template, kit, swab, and Sterivex controls) were included in the Illumina sequencing pool (30 μl), even if amplicons were not detected. Selected samples (see Table S1) were amplified using universal prokaryotic primers 515F-Y (35) and 926R (36) according to the method described above with annealing temperature at 50°C. Pooled 16S rRNA gene amplicons were excised from an agarose gel and purified using the Wizard SV Gel and PCR Clean-Up system (Promega, WI, USA). A 5-pM library containing 5% PhiX Control v3 (Illumina Canada, NB, Canada) was sequenced on a MiSeq (Illumina, CA, USA) using a 2 × 250-cycle MiSeq reagent kit v2 (Illumina Canada).

### Illumina sequence analysis.

All MiSeq reads were demultiplexed using MiSeq Reporter software (version 2.5.0.5, Illumina) and analyzed using Quantitative Insights Into Microbial Ecology 2 (QIIME2; release 2019.7) (37). The sequences were truncated to the shared V4 region by trimming to 515F/805R primer binding sites using Cutadapt (cutadapt trim-paired, version 2.4) (38). DADA2 (DADA2 denoise-paired, version 1.6) (39) was then used to denoise and dereplicate sequencing data and merge paired-end reads to generate an amplicon sequence variant (ASV) table. Taxonomy was assigned to ASVs using a Naive Bayes classifier (feature-classifier classify-sklearn) pretrained with SILVA database release 132 (40) with reference sequences trimmed to the target region (515F and 805R) to improve taxonomic classification (41). Bubble plots showing taxonomy profiles were created using the ggplot2 package (42) in R v.3.4.0 using ASV tables generated by QIIME2. Controls with fewer than 500 reads were excluded from the rarefied analysis.

### Phospholipid fatty acid extraction and analysis.

Lipids were extracted twice from 40 to 50 g of lyophilized bentonite core samples with a modified Bligh and Dyer protocol (43) using a 1:2:0.8 ratio of dichloromethane/methanol/phosphate buffer. Lipids were separated using silica gel chromatography. The polar fraction was subjected to methanolysis under mildly alkaline conditions to convert all phospholipids to fatty acid methyl esters (FAMEs). FAMEs were purified using secondary silica gel chromatography and identified on an Agilent 6890N gas chromatograph equipped with a DB5-MS column (30 m, 0.25 mm, 0.25-μm film thickness) coupled with an Agilent 5973 inert mass selective detector (quadruple). The splitless injection port temperature was 300°C with a column flow of 1.4 ml/min. The temperature program was as follows: oven hold at 50°C for 1 min, ramp 20°C/min to 120°C, ramp 4°C/min to 160°C, ramp 8°C/min to 300°C, and then hold 5 min at 300°C. Peaks were identified using retention times and molecular weights in comparison to the National Institute of Standards and Technology MS database and bacterial reference standards (Bacterial Acid Methyl Ester mix and Supelco 37 Component FAME Mix; Sigma-Aldrich).

### Data availability.

All sequences were deposited into European Nucleotide Archive (https://www.ebi.ac.uk/ena) with study accession number PRJEB24856. An unrarefied ASV table including all samples (ASV Table 1) is available at https://doi.org/10.5281/zenodo.3530762. A rarefied ASV table including only bentonite samples (ASV Table 2) is available at https://doi.org/10.5281/zenodo.3530764.

10.1128/mSphere.00601-19.5 TABLE S5 Comparison of sequencing reads and ASV counts for controls. For each control group, a various number of replicates (n) were available. Standard deviation (SD) was determined for all groups. Download Table S5, PDF file, 0.1 MB. Copyright © 2019 Engel et al. 2019 Engel et al. This content is distributed under the terms of the Creative Commons Attribution 4.0 International license.

10.1128/mSphere.00601-19.10 TEXT S1 DNA contamination controls. Download Text S1, PDF file, 0.1 MB. Copyright © 2019 Engel et al. 2019 Engel et al. This content is distributed under the terms of the Creative Commons Attribution 4.0 International license.
