# Supplementary material for: Stability of Microbial Community Profiles Associated with Compacted Bentonite from the Grimsel Underground Research Laboratory
Source: mSphere. 2019 Dec 18;4(6):e00601-19. doi: 10.1128/mSphere.00601-19 (PMC6920512; doi:10.1128/mSphere.00601-19)
Supplement: TABLE S1 [file mSphere.00601-19-st001.pdf]

Table S1.

Sample #	Module #	Sampling date	Control type	Control description	Sampling material	Extraction kit	gDNA (ng/µl)	Amplicon PCR2	Primer pair 1	Primer pair 2	PLFA analysis
Controls											
NTC1	-	-	No-template control	Amplified for 35 cycles	-	-	-	No	Pro341F/ Pro805R	-	-
NTC2	-	-	No-template control	Amplified for 50 cycles	-	-	-	No	Pro341F/ Pro805R	-	-
NTC3	-	-	No-template control	Amplified for 15 cycles	-	-	-	No	Pro341F/ Pro805R	-	-
CTRL1	-	-	Kit control	Simulated DNA extraction from kit buffer instead of a sample	-	PS	-	No	Pro341F/ Pro805R	-	-
CTRL2	-	-	Kit control	Simulated DNA extraction from kit buffer instead of a sample	-	PS	-	No	Pro341F/ Pro805R	-	-
CTRL3	-	-	Kit control	Simulated DNA extraction from kit buffer instead of a sample	-	PS	-	No	Pro341F/ Pro805R	-	-
CTRL6	-	-	Kit control	Simulated DNA extraction from kit buffer instead of a sample	-	PS	-	No	Pro341F/ Pro805R	-	-
CTRL7	-	-	Kit control	Simulated DNA extraction from kit buffer instead of a sample	-	PS	-	No	Pro341F/ Pro805R	-	-
CTRL8	-	-	Kit control	Simulated DNA extraction from kit buffer instead of a sample	-	PM	-	No	Pro341F/ Pro805R	-	-
CTRL9	-	-	Kit control	Simulated DNA extraction from kit buffer instead of a sample	-	PM	-	No	Pro341F/ Pro805R	-	-
1a	-	2015-Nov-10	Swab control	Simulated DNA extraction from an unused swab	Swab	PS	BDL	No	Pro341F/ Pro805R	-	-
1b	-	2015-Nov-10	Swab control	Simulated DNA extraction from an unused swab	Swab	PS	BDL	Yes	Pro341F/ Pro805R	-	-
1c	-	2015-Nov-10	Swab control	Simulated DNA extraction from an unused swab	Swab	PS	BDL	Yes	Pro341F/ Pro805R	-	-
Swab	-	2015-Nov-10	Swab control	Simulated DNA extraction from an unused swab	Swab	PS	BDL	Yes	Pro341F/ Pro805R	-	-
2a	-	2015-Nov-10	Glovebox control	Swab of glovebox, inside right wall	Swab	PS	BDL	No	Pro341F/ Pro805R	-	-
2b	-	2015-Nov-10	Glovebox control	Swab of glovebox, inside of front window	Swab	PS	BDL	Yes	Pro341F/ Pro805R	-	-
2c	-	2015-Nov-10	Glovebox control	Swab of glovebox, working surface	Swab	PS	BDL	Yes	Pro341F/ Pro805R	-	-
11a	-	2015-Nov-12	Glovebox control	Swab of glovebox, inside right wall	Swab	PS	BDL	No	Pro341F/ Pro805R	-	-
11b	-	2015-Nov-12	Glovebox control	Swab of glovebox, inside of front window	Swab	PS	BDL	No	Pro341F/ Pro805R	-	-
11c	-	2015-Nov-12	Glovebox control	Swab of glovebox gloves (without disposable glove on top)	Swab	PS	BDL	Yes	Pro341F/ Pro805R	-	-
42	-	2015-Oct-21	Sterivex control	Simulated DNA extraction from an unused Sterivex filter	Sterivex filter	PS	BDL	No	Pro341F/ Pro805R	-	-
Process blank 1	-	-	PLFA blank	Simulated PLFA extraction wihtout sample	-	-	-	-	-	-	Yes
Process blank 2	-	-	PLFA blank	Simulated PLFA extraction wihtout sample	-	-	-	-	-	-	Yes

Sample #	Module #	Sampling date	Sample location 1	Sample location 2	Sample description	Sampling material	Extraction kit	gDNA (ng/µl)	Amplicon PCR2	Primer pair 1	Primer pair 2	PLFA analysis
Samples												
4	1A	2015-Nov-10	Shipping flask	-	Liquid from inside of the shipping flask	Liquid	PS	1.4	Yes	Pro341F/ Pro805R	-	-
5a	1A	2015-Nov-10	Case	Fig. 1, image A	Swab of 5 permeable holes in the case, accessed from the outside	Swab	PS	BDL	Yes	Pro341F/ Pro805R	-	-
5b	1A	2015-Nov-10	Case	Fig. 1, image A	Swab of 5 permeable holes in the case, accessed from the outside	Swab	PS	BDL	Yes	Pro341F/ Pro805R	-	-
6a	1A	2015-Nov-10	Case	Fig. 1, image A	Swab of the case, outside surface	Swab	PS	BDL	Yes	Pro341F/ Pro805R	-	-
6b	1A	2015-Nov-10	Case	Fig. 1, image A	Swab of the case, outside surface	Swab	PS	BDL	Yes	Pro341F/ Pro805R	-	-
7	1A	2015-Nov-10	Case	Fig. S2, image B	Piece of black deposit from the inside of the lid	Solid	PS	0.09	Yes	Pro341F/ Pro805R	-	-
8a	1A	2015-Nov-10	Case	-	Swab of the case, inside, top (mix of black deposit and wet bentonite)	Swab	PS	0.05	Yes	Pro341F/ Pro805R	-	-
8b	1A	2015-Nov-10	Case	-	Swab of the case, inside, bottom (mix of black deposit and wet bentonite)	Swab	PS	BDL	Yes	Pro341F/ Pro805R	-	-
9a	1A	2015-Nov-11	Filter	Fig. 1, image C	Swab of the filter, outside surface	Swab	PS	BDL	Yes	Pro341F/ Pro805R	-	-
9b	1A	2015-Nov-11	Filter	Fig. 1, image C	Swab of the filter, outside surface	Swab	PS	BDL	Yes	Pro341F/ Pro805R	-	-
10a	1A	2015-Nov-11	Filter	Fig S2, image E	Swab of the filter, inside surface	Swab	PS	BDL	Yes	Pro341F/ Pro805R	-	-
10b	1A	2015-Nov-11	Filter	Fig S2, image E	Swab of the filter, inside surface	Swab	PS	BDL	Yes	Pro341F/ Pro805R	-	-
26	1A	2015-Nov-11	Bentonite, outer layer	Fig. 1, image D	Composite sample of bentonite from sections 1 to 4	Bentonite	PM/ PS	0.15/ BDL	Yes/ No	Pro341F/ Pro805R	-	Yes
27	1A	2015-Nov-11	Bentonite, inner layer	Fig. 1, image D	Composite sample of bentonite from sections 1 to 4	Bentonite	PM/ PS	0.05/ BDL	Yes/ Yes	Pro341F/ Pro805R	515F-Y/ 926R	Yes
28	1A	2015-Nov-11	Bentonite, outer layer S5	Fig. 1, image D	Bentonite from section 5	Bentonite	PM/ PS	0.08/ BDL	Yes/ Yes	Pro341F/ Pro805R	515F-Y/ 926R	-
29	1A	2015-Nov-11	Bentonite, inner layer S5	Fig. 1, image D	Bentonite from section 5	Bentonite	PM/ PS	0.07/ BDL	Yes/ No	Pro341F/ Pro805R	515F-Y/ 926R	Yes
14	2A	2015-Nov-12	Shipping flask	-	Liquid in shipping flask	Liquid	PS	1.31	Yes	Pro341F/ Pro805R	-	-
15a	2A	2015-Nov-12	Case	Fig. 1 A	Swab of 5 permeable holes in the case, accessed from the outside	Swab	PS	BDL	Yes	Pro341F/ Pro805R	-	-
15b	2A	2015-Nov-12	Case	Fig. 1 A	Swab of 5 permeable holes in the case, accessed from the outside	Swab	PS	BDL	Yes	Pro341F/ Pro805R	-	-
16a	2A	2015-Nov-12	Case	Fig. 1 A	Swab of the case, outside surface	Swab	PS	0.15	Yes	Pro341F/ Pro805R	-	-
16b	2A	2015-Nov-12	Case	Fig. 1 A	Swab of the case, outside surface	Swab	PS	0.04	Yes	Pro341F/ Pro805R	-	-
17	2A	2015-Nov-12	Case	Fig. S2, image B	Piece of black deposit from the inside of the lid	Solid	PS	0.04	Yes	Pro341F/ Pro805R	-	-
18a	2A	2015-Nov-12	Case	-	Swab of the case, inside, top	Swab	PS	BDL	Yes	Pro341F/ Pro805R	-	-
18b	2A	2015-Nov-12	Case	-	Swab of the case, inside, bottom	Swab	PS	BDL	Yes	Pro341F/ Pro805R	-	-
19a	2A	2015-Nov-12	Filter	Fig. 1, image C	Swab of the filter, outside surface	Swab	PS	BDL	Yes	Pro341F/ Pro805R	-	-
19b	2A	2015-Nov-12	Filter	Fig. 1, image C	Swab of the filter, outside surface	Swab	PS	BDL	Yes	Pro341F/ Pro805R	-	-
20a	2A	2015-Nov-12	Filter	Fig S2, image E	Swab of the filter, inside surface	Swab	PS	BDL	Yes	Pro341F/ Pro805R	-	-
20b	2A	2015-Nov-12	Filter	Fig S2, image E	Swab of the filter, inside surface	Swab	PS	BDL	Yes	Pro341F/ Pro805R	-	-
35	2A	2015-Nov-13	Bentonite, outer layer	Fig. 1, image D	Composite sample of bentonite from sections 1 to 4	Bentonite	PM/ PS	0.06/ BDL	Yes/ No	Pro341F/ Pro805R	-	Yes
36	2A	2015-Nov-13	Bentonite, inner layer	Fig. 1, image D	Composite sample of bentonite from sections 1 to 4	Bentonite	PM/ PS	0.07/ BDL	Yes/ No	Pro341F/ Pro805R	515F-Y/ 926R	Yes
37	2A	2015-Nov-13	Bentonite, outer layer S5	Fig. 1, image D	Bentonite from section 5	Bentonite	PM	0.05 /BDL	Yes/ No	Pro341F/ Pro805R	515F-Y/ 926R	-
38	2A	2015-Nov-13	Bentonite, inner layer S5	Fig. 1, image D	Bentonite from section 5	Bentonite	PM	0.05/ BDL	Yes	Pro341F/ Pro805R	515F-Y/ 926R	Yes
43	-	2015-Oct-21	Borehole fluid	-	Sterivex filtered fluid from borehole 13.001 (volume unknown)	Sterivex filter	PS	2.49	Yes	Pro341F/ Pro805R	-	Yes
47	-	2015-Jun-24	Borehole fluid	-	Sterivex filtered fluid directly from borehole 85.003 (1200 ml filtered)	Sterivex filter	PS	BDL	Yes	Pro341F/ Pro805R	-	-
M6	-	-	Bentonite	-	Wyoming MX-80 bentonite, uncompacted, powder	Bentonite	PM	0.03	Yes	-	515F-Y/ 926R	-
M7	-	-	Bentonite	-	Wyoming MX-80 bentonite, uncompacted, pellets	Bentonite	PM	0.04	Yes	-	515F-Y/ 926R	-
