# Supplementary figures and images for: Stability of Microbial Community Profiles Associated with Compacted Bentonite from the Grimsel Underground Research Laboratory
Source: mSphere. 2019 Dec 18;4(6):e00601-19. doi: 10.1128/mSphere.00601-19 (PMC6920512; doi:10.1128/mSphere.00601-19)

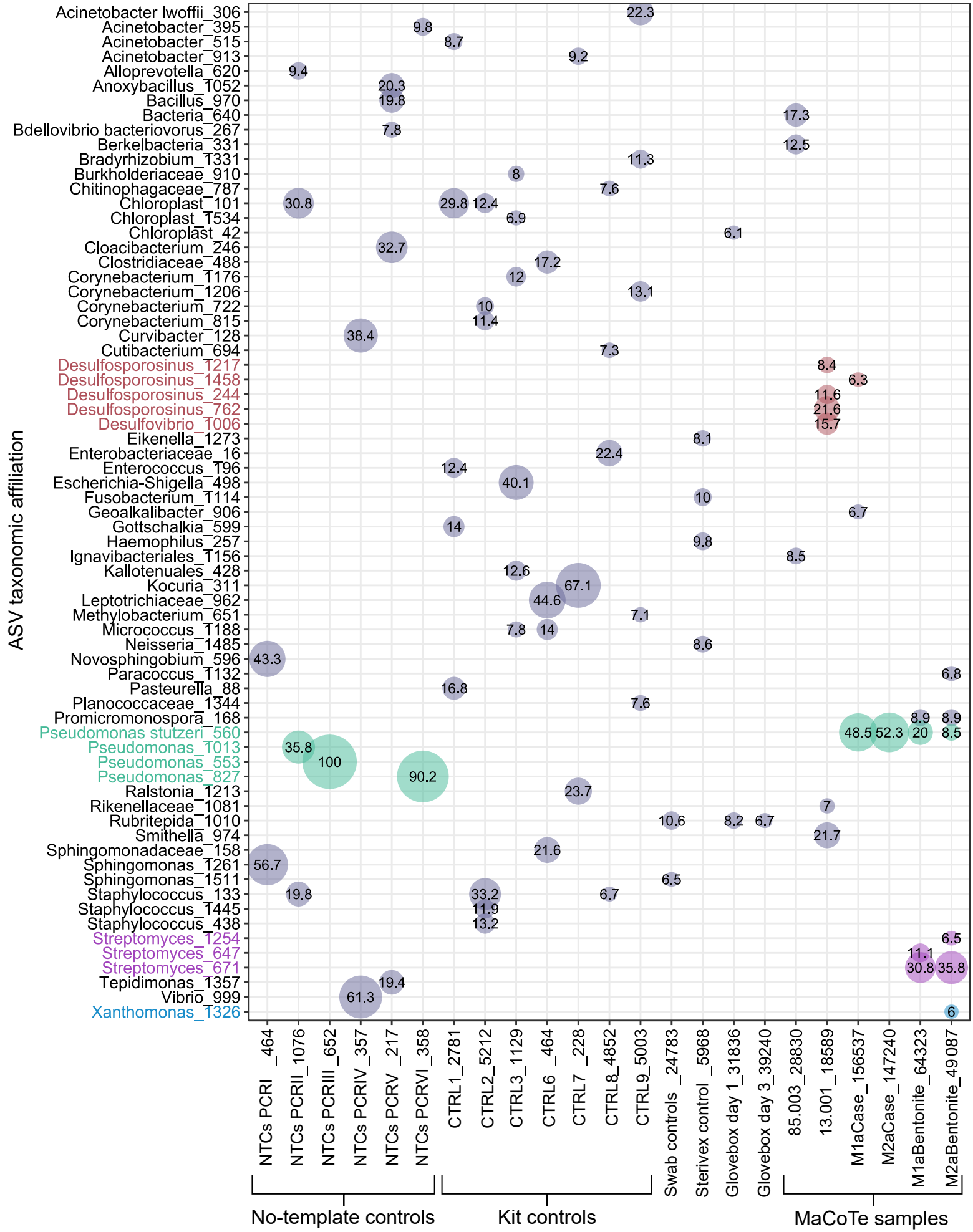

Supplement: FIG S1 [file mSphere.00601-19-sf001.pdf]

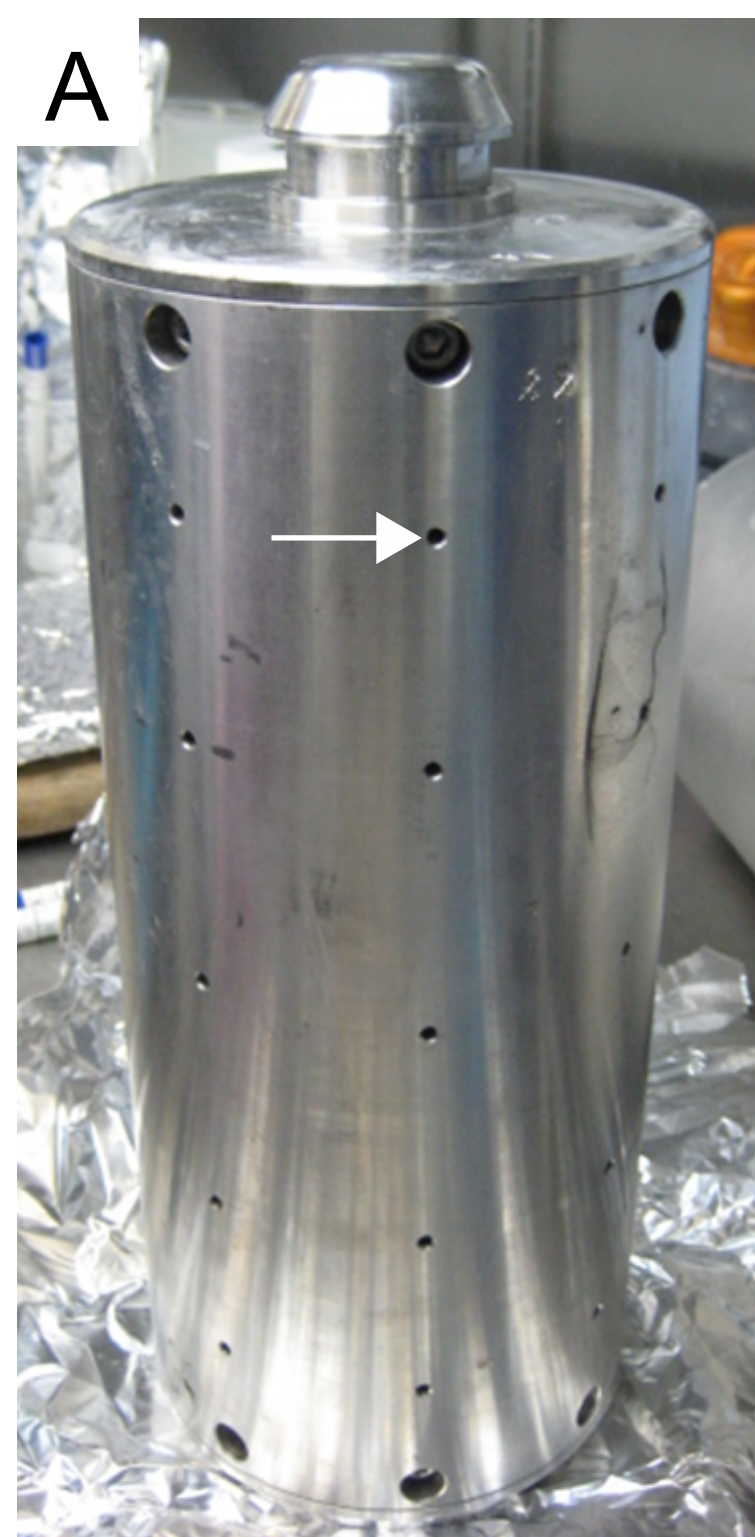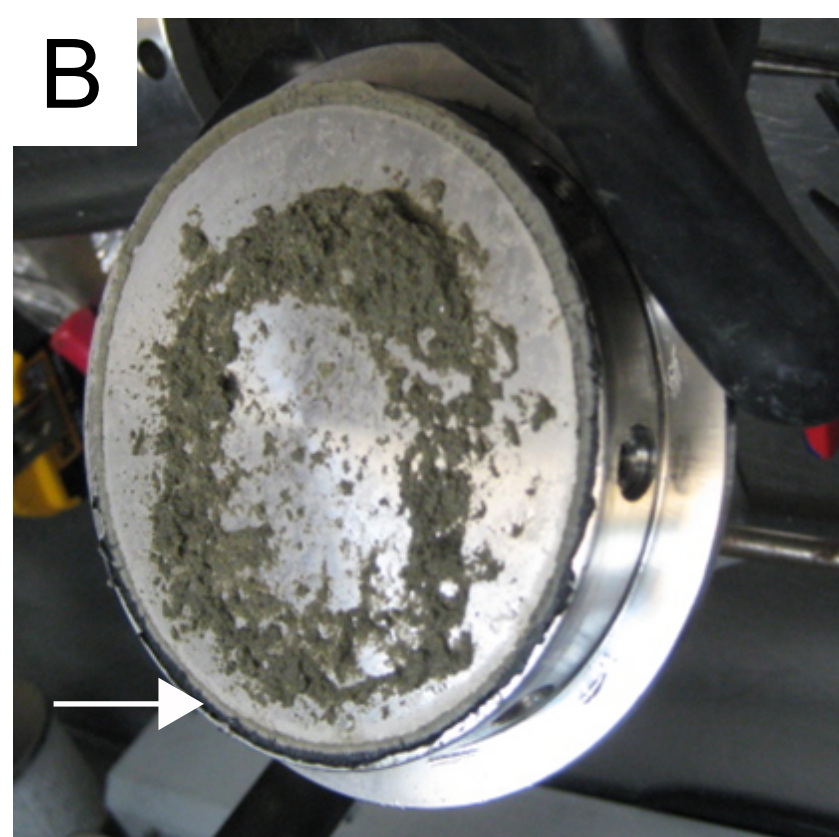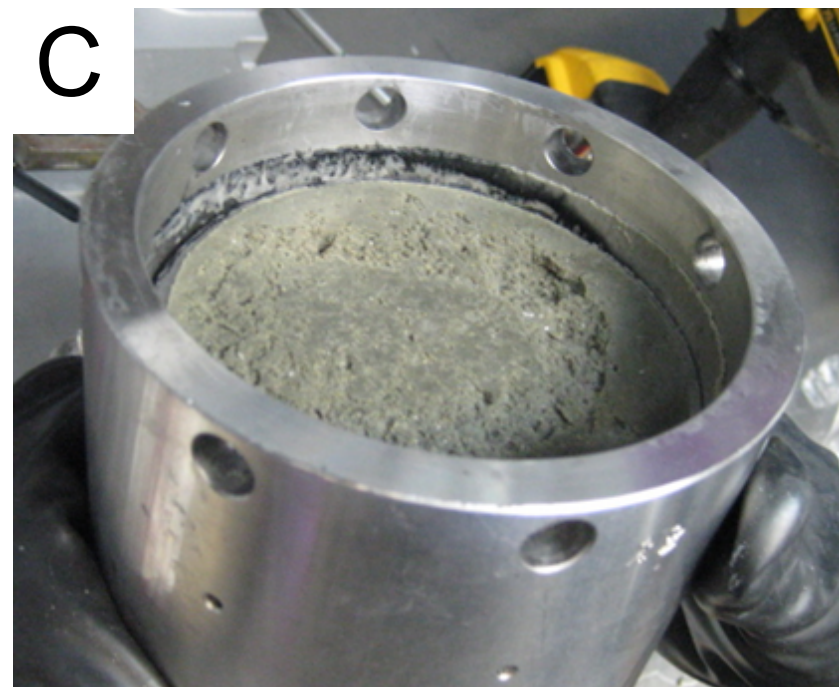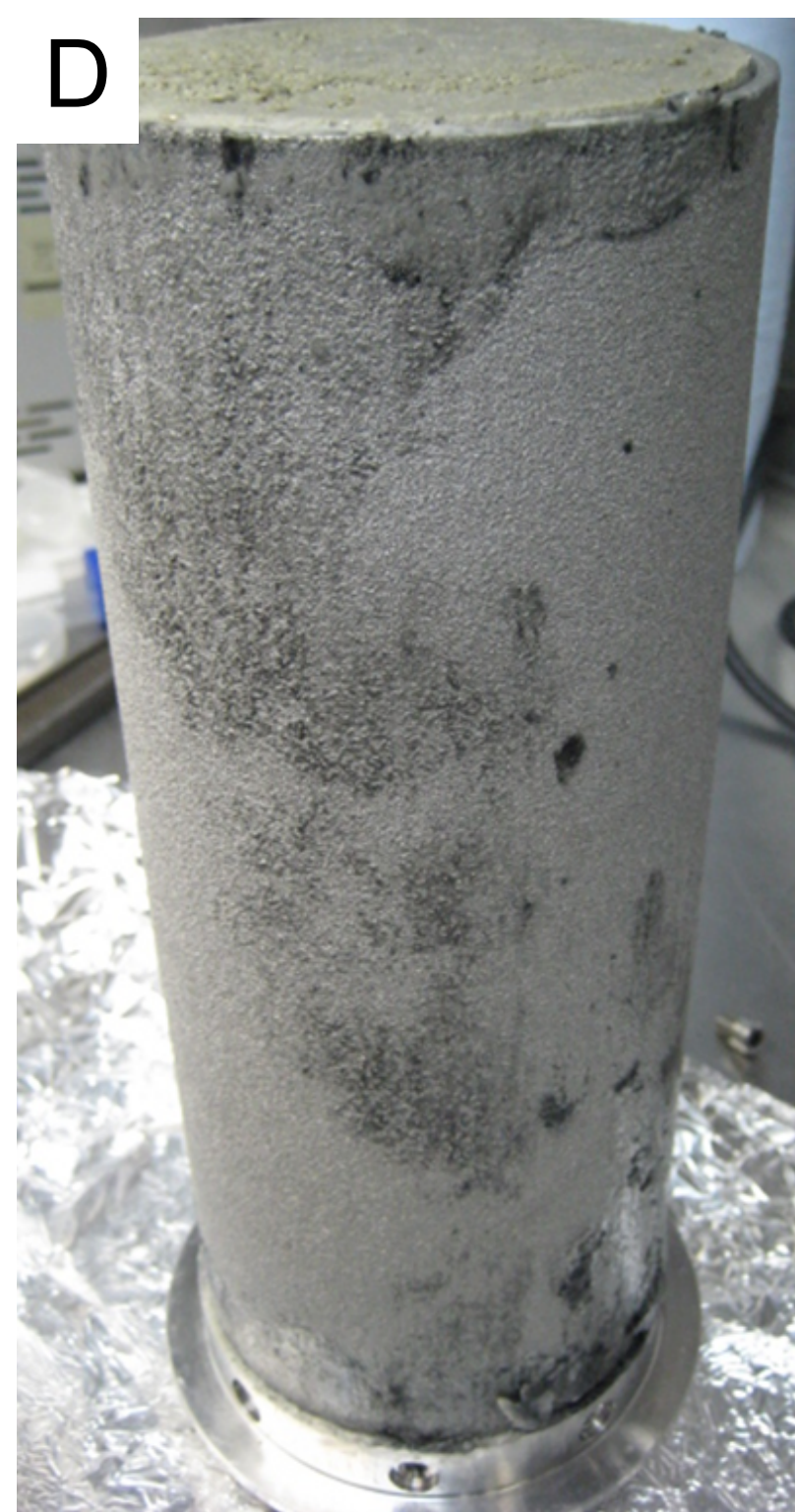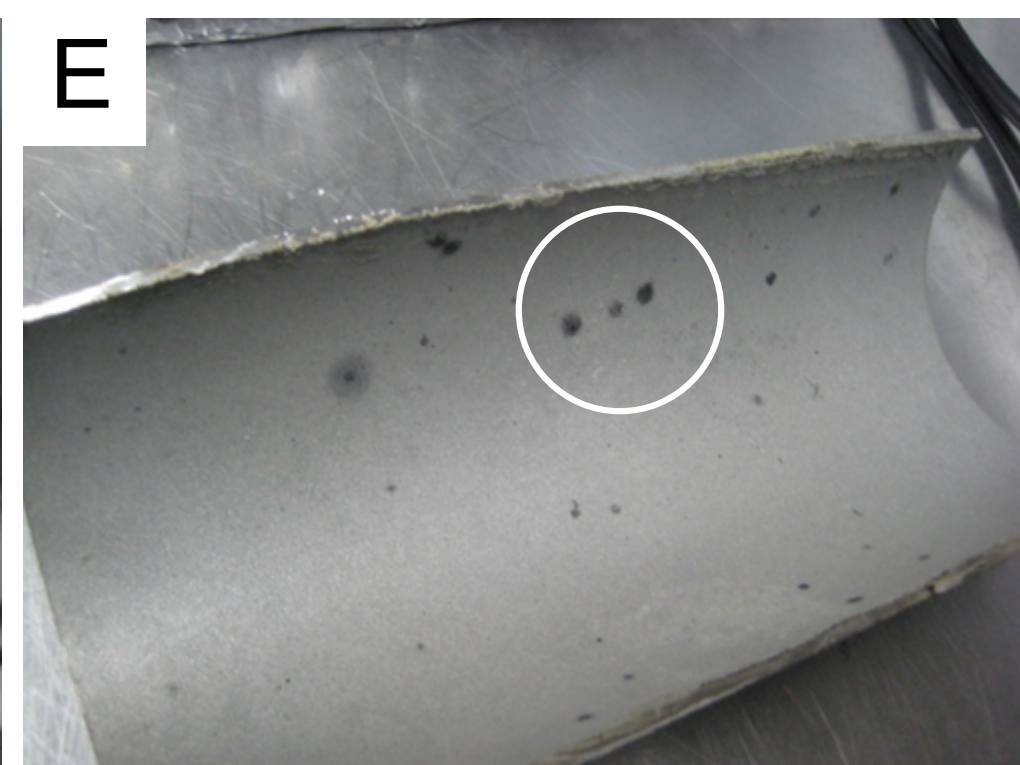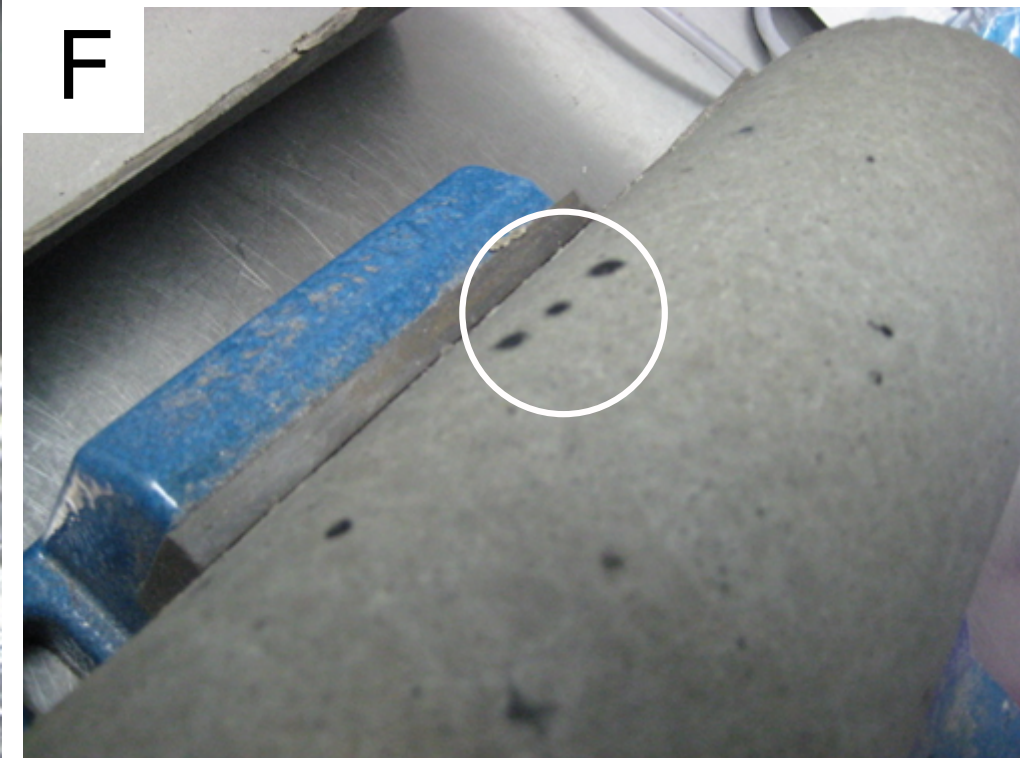

Supplement: FIG S2 [file mSphere.00601-19-sf002.pdf]

A

ASV taxonomic affiliation

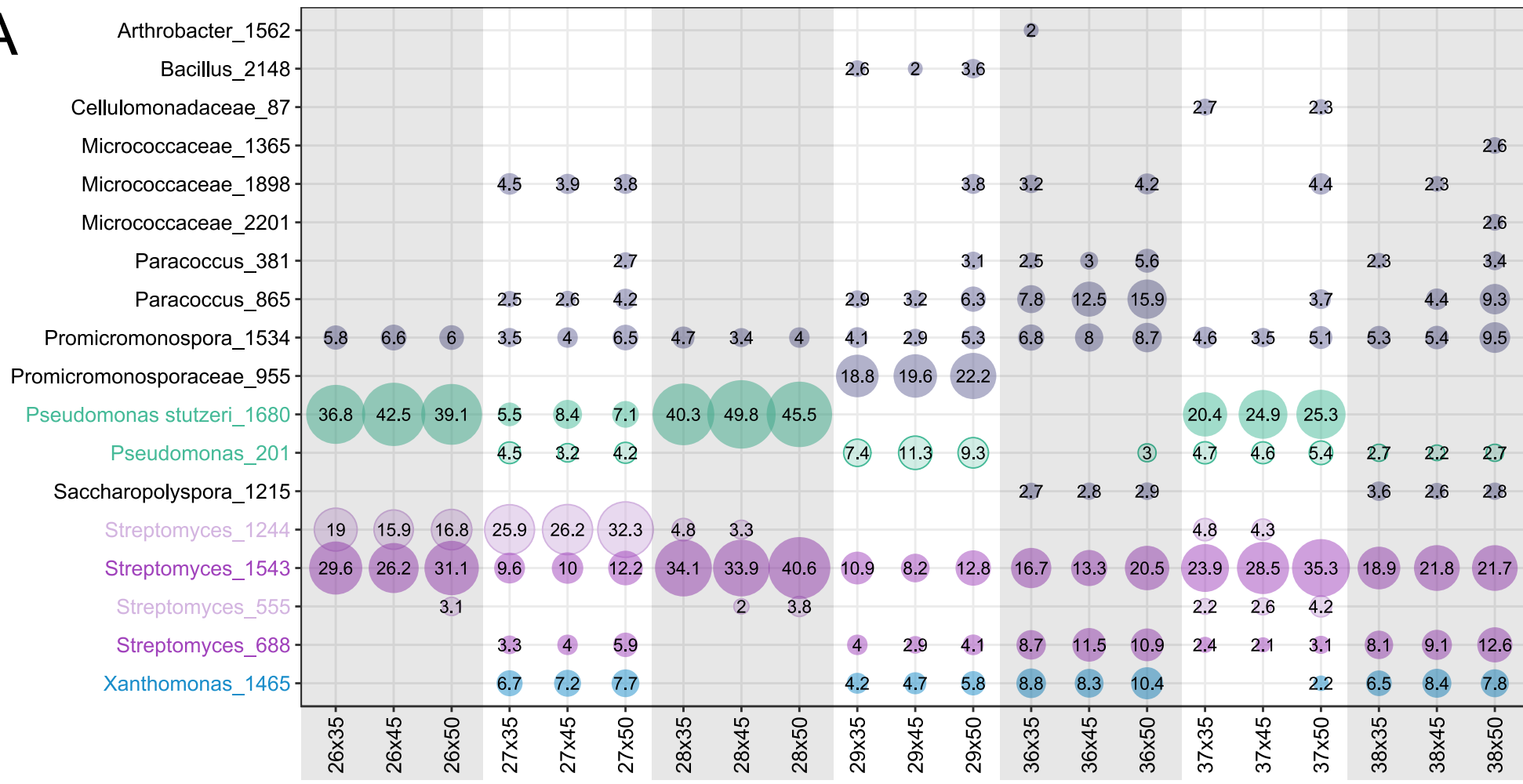

B

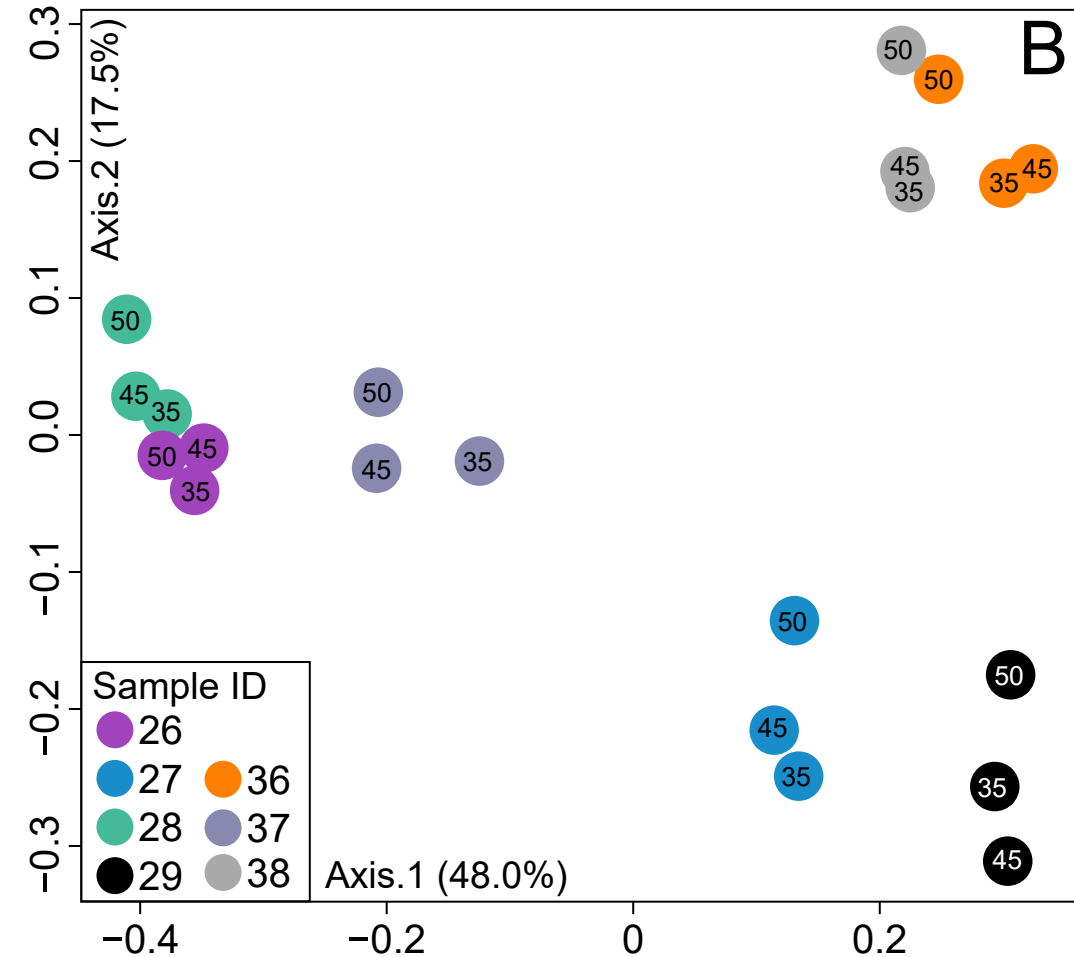

Supplement: FIG S3 [file mSphere.00601-19-sf003.pdf]
