# Supplementary material for: Stability of Microbial Community Profiles Associated with Compacted Bentonite from the Grimsel Underground Research Laboratory
Source: mSphere. 2019 Dec 18;4(6):e00601-19. doi: 10.1128/mSphere.00601-19 (PMC6920512; doi:10.1128/mSphere.00601-19)
Supplement: TABLE S2 [file mSphere.00601-19-st002.pdf]

Table S2.

**Bentonite**	**n**	**Average ASV count**	**SD**	**Average ASV count (≥1%)**	**SD**
Module 1A outer layer	4	9	3	8	2
Module 1A inner layer	4	24	10	18	6
Module 2A outer layer	4	18	6	11	3
Module 2A inner layer	4	28	4	18	3
