# Supplementary material for: Stability of Microbial Community Profiles Associated with Compacted Bentonite from the Grimsel Underground Research Laboratory
Source: mSphere. 2019 Dec 18;4(6):e00601-19. doi: 10.1128/mSphere.00601-19 (PMC6920512; doi:10.1128/mSphere.00601-19)
Supplement: FIG S4 [file mSphere.00601-19-sf004.pdf]

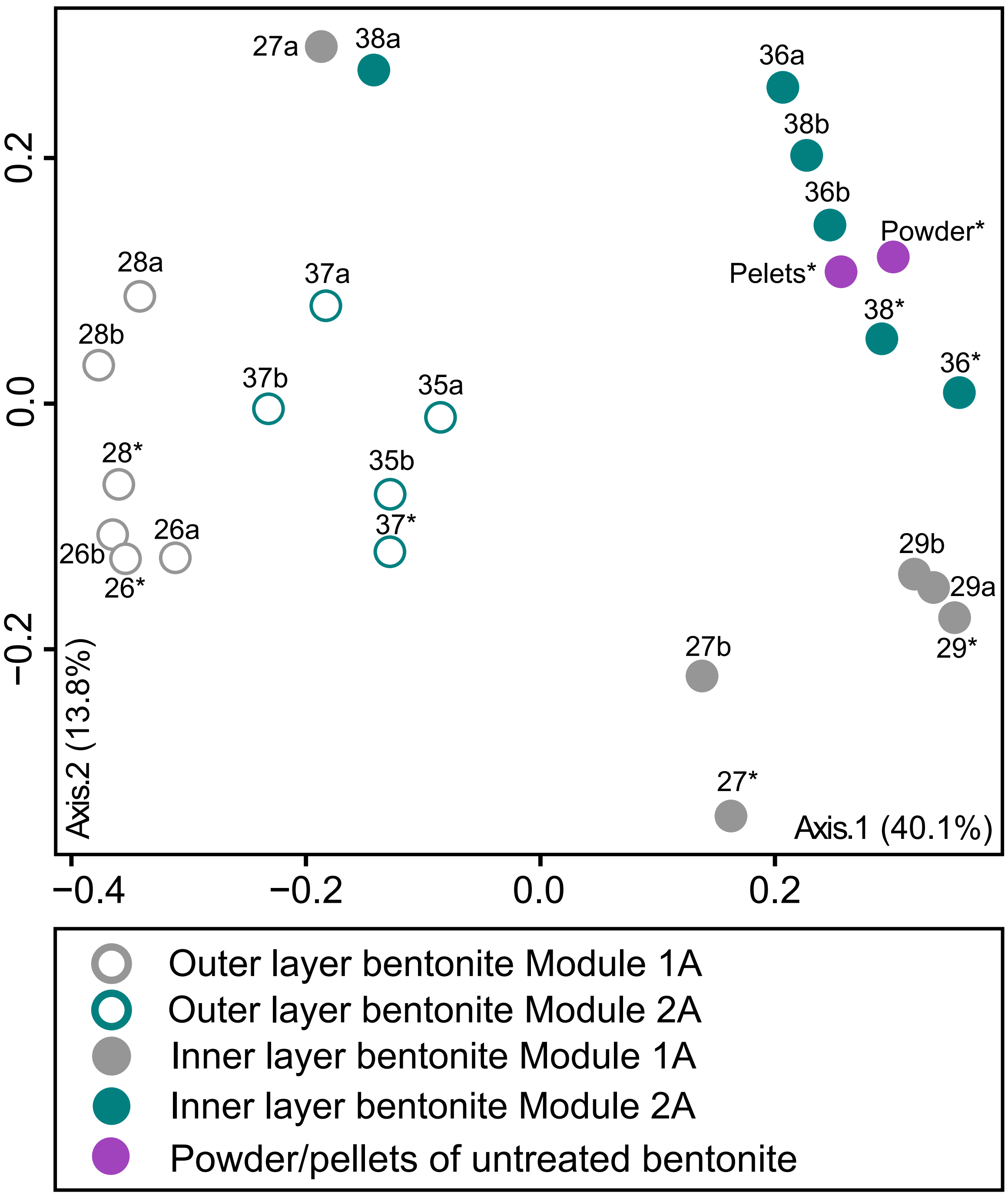

27a
38a
36a
38b
36b
Powder*
Pelets*
38*
36*
28a
37a
28b
37b
35a
28*
35b
37*
26a
26b
26*
29b
29a
29*
27b
27*
0.2
0.0
−0.2
−0.4
0.2
Axis.2 (13.8%)
Axis.1 (40.1%)
Outer layer bentonite Module 1A
Outer layer bentonite Module 2A
Inner layer bentonite Module 1A
Inner layer bentonite Module 2A
Powder/pellets of untreated bentonite
