# Supplementary material for: Stability of Microbial Community Profiles Associated with Compacted Bentonite from the Grimsel Underground Research Laboratory
Source: mSphere. 2019 Dec 18;4(6):e00601-19. doi: 10.1128/mSphere.00601-19 (PMC6920512; doi:10.1128/mSphere.00601-19)
Supplement: TABLE S3 [file mSphere.00601-19-st003.pdf]

Table S3.

**Sample**	**PLFA (pmol)**	**PLFA (pmol/g)**	**Predicted abundance (cells/g)**
Process blank 1	463	NA	NA
Process blank 2	361	NA	NA
**Module 1A bentonite**			
Outer layer	5563	140	3E+06
Inner layer	4056	81	2E+06
Inner layer Section 5	4474	86	2E+06
**Module 2A bentonite**			
Outer layer	3556	63	1E+06
Inner layer	4043	70	1E+06
Inner layer Section 5	4574	80	2E+06
**Borehole 13.001 fluid**	**(pmol)**	**(pmol/ml)**	**(cells/ml)**
13.001a	609	5	2E+05
13.001b	715	0.75	3E+04
