# Supplementary material for: Stability of Microbial Community Profiles Associated with Compacted Bentonite from the Grimsel Underground Research Laboratory
Source: mSphere. 2019 Dec 18;4(6):e00601-19. doi: 10.1128/mSphere.00601-19 (PMC6920512; doi:10.1128/mSphere.00601-19)
Supplement: TABLE S4 [file mSphere.00601-19-st004.pdf]

Table S4.

**ASV ID**	**Description**	**Max score**	**Total score**	**Query cover (%)**	**E value**	**Identity (%)**
#671	*Streptomyces iconiensis*	464	464	100%	9e-131	100%
	Streptomyces chumphonensis	464	464	100%	9e-131	100%
