# Supplementary material for: Stability of Microbial Community Profiles Associated with Compacted Bentonite from the Grimsel Underground Research Laboratory
Source: mSphere. 2019 Dec 18;4(6):e00601-19. doi: 10.1128/mSphere.00601-19 (PMC6920512; doi:10.1128/mSphere.00601-19)
Supplement: TABLE S5 [file mSphere.00601-19-st005.pdf]

Table S5.

**Controls**	**n**	**Average read count**	**SD**	**Average ASV count**	**SD**
Kit control (PowerMax)	2	4,928	107	23	0
Kit control (PowerSoil)	5	1,963	2,072	7	3
Unused swab control	4	6,196	4,986	24	18
NTC1 (35 cycles)	6	102	74	2	2
NTC2 (nested, 50 cycles)	6	418	210	2	1
NTC3 (15 cycles)	4	0	1	0	1
