# Supplementary material for: Stability of Microbial Community Profiles Associated with Compacted Bentonite from the Grimsel Underground Research Laboratory
Source: mSphere. 2019 Dec 18;4(6):e00601-19. doi: 10.1128/mSphere.00601-19 (PMC6920512; doi:10.1128/mSphere.00601-19)
Supplement: TEXT S1 [file mSphere.00601-19-s0001.pdf]

DNA contamination controls

Several controls were included in the analysis to identify contaminating DNA from reagents or materials. Controls for the DNA isolation kit ("kit control"; simulated DNA extraction from kit buffer instead of a sample) were performed with each batch of extractions. "Swab controls" (simulated DNA extraction from an unused swab), and "Sterivex control" (simulated DNA extraction from an unused Sterivex filter) were included as well. No-template controls (NTCs) were prepared with each batch of 16S rRNA gene amplification. Along with samples, NTCs were amplified for 35 (NTC1), 50 (NTC2), or 15 cycles (NTC3) in six different PCR amplifications (PCR I to PCR VI), involving six independent PCR master mixes. None of the controls showed a visible 16S rRNA gene amplicon in agarose gels stained with GelRed (data not shown). The read counts for unnested PCR reagent controls (NTC1, NTC3) were very low, with 102 and 0 (Table S5). Average read counts for nested controls (NTC2) increased to 418 reads (Table S5).

When comparing all six master mix controls, no dominant ASV could be identified that was present in all of them (Figure S1). The read counts in kit and swab controls were on average 13 times higher than in nested NTCs, but deviation was very high (Table S5). Beta diversity measures using principal coordinate analysis (PCoA) ordinations showed that bacterial communities in the controls did not differ detectably in the first two dimensions of the ordination from all samples (panel A in Figure 3). Samples that are closer together with the controls on the ordination have communities that are likely more similar to one another. Due to low DNA concentration in those samples, the PCR might have only amplified background contamination but no sample-specific DNA. However, controls separate well from bentonite and filter samples as well as the majority of case samples.

Contamination from DNA extraction kit reagents were reported previously (1, 2), however, in five PowerSoil (CTRL1-3, 6, 7) and two PowerMax (CTRL8-9) extraction kit controls, we could not identify common taxa (Figure S1). ASVs affiliated with *Staphylococcus* were present in several extraction controls (Figure S1) and might be contaminants from kit reagents. ASVs associated with Pseudomonas were also identified in several controls, however, different ASVs were present in bentonite samples (Figure S1). Borehole fluid samples grouped closely with controls in the PCoA ordination (Figure 3). However, both replicate extractions yielded similar taxonomic profiles and dominant ASVs were absent from controls (Figure S1).

References

1. Salter SJ, Cox MJ, Turek EM, Calus ST, Cookson WO, Moffatt MF, Turner P, Parkhill J, Loman NJ, Walker AW. 2014. Reagent and laboratory contamination can critically impact sequence-based microbiome analyses. BMC Biol 12:87.
2. Glassing A, Dowd SE, Galandiuk S, Davis B, Chiodini RJ. 2016. Inherent bacterial DNA contamination of extraction and sequencing reagents may affect interpretation of microbiota in low bacterial biomass samples. Gut Pathog 8.
